# Plant-Origin Components: New Players to Combat Antibiotic Resistance in *Klebsiella pneumoniae*

**DOI:** 10.3390/ijms25042134

**Published:** 2024-02-10

**Authors:** Victor M. Luna-Pineda, Griselda Rodríguez-Martínez, Marcela Salazar-García, Mariana Romo-Castillo

**Affiliations:** 1 Laboratorio de Investigación en COVID-19, Hospital Infantil de México Federico Gómez, Ciudad de México 06720, Mexico; luna.pineda@hotmail.com (V.M.L.-P.); griseldargzmtz123@gmail.com (G.R.-M.); 2 Laboratorio de Investigación en Inmunología y Proteómica, Hospital Infantil de México Federico Gómez, Ciudad de México 06720, Mexico; 3 Departamento de Investigación Biomédica, Hospital Infantil de México Federico Gómez, Ciudad de México 06720, Mexico; 2016msg.ciencias@gmail.com; 4 IxM/CONAHCYT-HIMFG, Laboratorio de Investigación en Inmunología y Proteómica, Hospital Infantil de México Federico Gómez, Ciudad de México 06720, Mexico

**Keywords:** *Klebsiella pneumoniae*, virulence factors, antibiotic resistance, plant-origin compounds, essential oils, plant extracts, treatment design

## Abstract

*Klebsiella pneumoniae* (Kpn) is an opportunistic pathogen that causes intrahospital complications such as pneumonia, liver abscesses, soft tissue infections, urinary infections, bacteraemia, and, in some cases, death. Since this bacterium has a higher frequency than other Gram-negative pathogens, it has become an important pathogen to the health sector. The adaptative genome of Kpn likely facilitates increased survival of the pathogen in diverse situations. Therefore, several studies have been focused on developing new molecules, synergistic formulations, and biomaterials that make it possible to combat and control infections with and dispersion of this pathogen. Note that the uncontrolled antibiotic administration that occurred during the pandemic led to the emergence of new multidrug-resistant strains, and scientists were challenged to overcome them. This review aims to compile the latest information on Kpn that generates intrahospital infections, specifically their pathogenicity-associated factors. Furthermore, it explains the natural-product-based treatments (extracts and essential oils) developed for Kpn infection and dispersion control.

## 1. Introduction: General Aspects of *Klebsiella pneumoniae*

**Figure 1:**
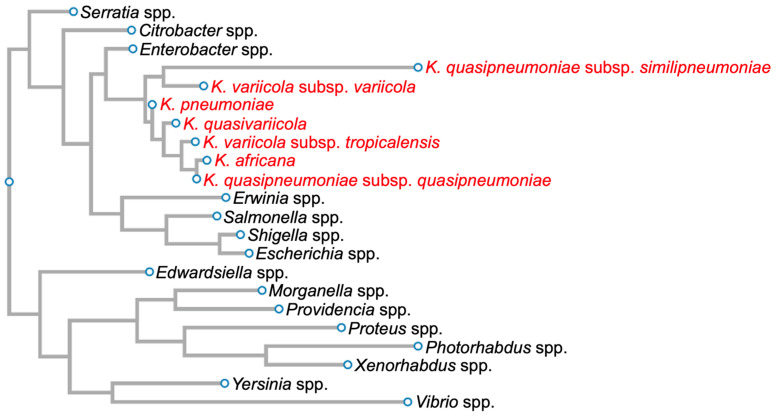
Phylogenetic tree of members of the family *Enterobacteriaceae* based on 16S rDNA sequences. Members of the *Klebsiella pneumoniae* species complex are in red. Diagram was generated for graphical information display using 16S rDNA sequences of *Serratia marcescens* strain Gol3 (ID: MT263018.1), *Citrobacter freundii* strain S12 (ID: MW879533.1), *Enterobacter cloacae* strain ATCC 13047 1 (ID: NR_102794.2), *Klebsiella quasipneumoniae* subsp. *similipneumoniae* strain CW-D 3 (ID: NR_132596.1), *Klebsiella variicola* strain 13450 (ID: CP026013.1), *Klebsiella pneumoniae* strain DSM 30104 (ID: NR_036794.1), *Klebsiella quasivariicola* strain KPN1705 (ID: OQ719747.1), *Klebsiella variicola* subsp. *tropicalensis* strain VITGAJ4 (ID: MT829337.1), *Klebsiella africana* strain SB5857 (ID: MK040622.1), *Klebsiella quasipneumoniae* subsp. *quasipneumoniae* strain 01A030 (ID: NR_134062.1), *Erwinia amylovora* strain S34 (ID: OP512541.1), *Salmonella enterica* strain 16OCT84 (ID: OQ581800.1), *Shigella dysenteriae* strain ATCC 13313 (ID: NR_026332.1), *Escherichia coli* strain Gol11 16S (ID: MT263026.1), *Edwardsiella tarda* strain KC-Pc-HB1(ID: CP023706.1), *Morganella morganii* subsp. *morganii* strain 229813 (ID: CP043955.1), *Providencia stuartii* strain ATCC 29914 (ID: NR_024848.1), *Proteus vulgaris* ATCC 29905 (ID: NR_115878.1), *Photorhabdus asymbiotica* strain ATCC 43951 (ID: Z76754.1), *Xenorhabdus nematophila* strain DSM3370 (ID: NR_119150.1), *Yersinia pestis* strain SCPM-O-B-6291 (ID: CP045163.1), and *Vibrio cholerae* strain RC782(ID: ON849168.1).

*Klebsiella pneumoniae* (Kpn) was first isolated by Carl Friedlander in 1882 from the lungs of dead pneumonia patients [1]. Hence, it was initially called *Friedlander’s bacillus*, but in 1886, it was renamed Kpn. This Gram-negative bacillus, an encapsulated, non-motile bacterium, is a lactose-fermenting, non-spore-forming facultative anaerobe that belongs to the *Enterobacteriaceae* family (Figure 1) [2].

Kpn is a *Klebsiella pneumoniae* species complex (KpSC) member, which includes strains that have the same biochemical profile but only share 90–96% identity between their genome sequences [3,4,5]. This complex contains seven phylogroups: *Klebsiella pneumoniae*, *Klebsiella quasipneumoniae* subsp. *quasipneumoniae*, *Klebsiella quasipneumoniae* subsp. *similipneumoniae*, *Klebsiella variicola* subsp. *variicola*, *Klebsiella variicola* subsp. *tropicalensis*, *Klebsiella quasivariicola*, and *Klebsiella africana* (Figure 1).

Although all these strains could be associated with human diseases, Kpn is mainly related to nosocomial infections [5]. Kpn strains are classified into three categories: classical (cKpn), hypervirulent (hvKpn), and multidrug-resistant (MDR-Kpn). cKpn strains are nosocomial strains that are generally found in immunocompromised patients and produce urinary tract, acute respiratory, and bloodstream infections [5]. cKpn strains are characterised by a non-hypermucoviscosity phenotype (“string test”) and lack excessive siderophores [6]. hvKpn strains are community-acquired strains that induce pyogenic liver abscess, meningitis, endophthalmitis, and necrotising fasciitis in diabetic and healthy people. These strains are characterised by a hypermucoviscosity phenotype and harbour excessive siderophores [7]. Finally, pyogenic liver abscesses, bloodstream infections, and urinary tract infections are associated with nosocomial and community MDR-Kpn strains that usually infect immunocompromised patients [8]. MDR-Kpn strains have a hypervirulent profile but no specific hypermucoviscosity-defined pattern [6,9]. MDR-Kpn and hvKpn strains were previously considered different clonal groups, but now these characteristics are deemed additive [10]. The high incidence of MDR-Kpn and hvKpn strains has earned Kpn a place on the World Health Organization (WHO) list as a “priority pathogen” since 2017 [11].

The complex biology of Kpn has made it difficult to control infections caused by this pathogen due to its high antimicrobial resistance and many virulence factors. That is why designing new therapies to combat Kpn infections has been a priority. Plants are a potential source for the design of new treatments [12]. Numerous biotic and abiotic stimuli, including certain pathogenic bacteria, cause plants to produce many secondary metabolites. These metabolites are employed in medicine to treat many illnesses, including infections involving bacteria. However, very few studies have demonstrated these metabolites’ antimicrobial potential, mechanism of action, and potential for developing new antibacterial therapies. This research aims to create a comprehensive list of the latest data regarding the application of plants to treat Kpn-induced illnesses.

## 2. Kpn Virulence Factors

**Figure 2:**
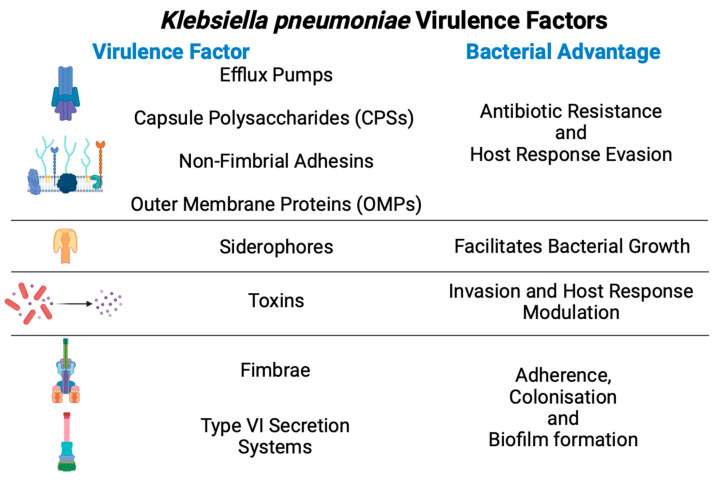
General virulence factors from *Klebsiella pneumoniae* and their advantages during infection.

Kpn has acquired many virulence factors to promote colonisation, evasion of host immune responses, and bacterial competition (Figure 2).

**Figure 3:**
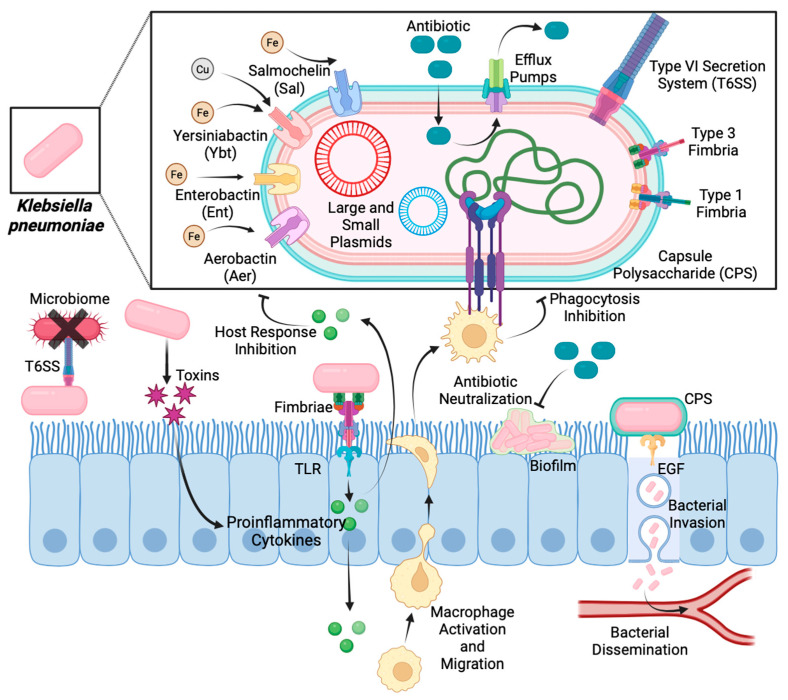
Mechanisms of action of virulence factors during *Klebsiella pneumoniae* infection.

Fimbriae, adhesins, and capsule polysaccharides (CPSs) from Kpn are the most representative virulence factors used to adhere to and colonise host cells. Fimbriae are hair-like appendages and are thin structures localised on cellular surfaces that allow Kpn adherence and colonisation. Fimbria type 1 is exclusively expressed in the urinary tract, favouring adhesion to the bladder [13]. In contrast, fimbria type 3 is expressed in kidney and lung cells for adherence and biofilm formation. Additionally, fimbriae of both types are associated with biofilm formation on abiotic surfaces such as those of catheters (Figure 3) [14,15].

During colonisation, capsule polysaccharides (CPSs) from Kpn are a physical barrier and promote host immune evasion (Figure 3). CPSs can block phagocytosis via IL-36 and act as a barrier against antibacterial peptides [16]. CPSs also activate the EGF–PI3K–Akt pathway, inducing cytoskeleton rearrangement in host cells and allowing the bacterium to translocate [17]. CPS types have a substantial variation, but Kpn strains possess at least two CPSs, KL106 and KL107 [18].

Outer membrane proteins (OMPs) are implicated in the modulation of immune response and antibiotic resistance as an essential factor. In Kpn infection, OmpA activates Toll-like receptor (TLR) 2, and OmpK36 contributes to resisting phagocytosis by neutrophils and macrophages [19,20,21]. On the other hand, OmpK35 and OmpK26 are associated with the efflux of antibiotics such as carbapenem [22].

Furthermore, lipopolysaccharide (LPS) in Kpn comprises an O antigen, lipid A, and an oligosaccharide core in lipid rafts. LPS protects against complement-mediated lysis and activates TLR4 [23].

Despite metal ions being critical elements required for many bacterial metabolic processes, the extraintestinal environment is deficient in ions such as iron and copper [24,25]. Bacteria have developed small iron-binding molecules, named siderophores, to bind and transport ions from the host cell, promoting bacterial growth [26,27]. The main Kpn siderophores are Enterobactin, Yersiniabactin, Salmochelin, and Aerobactin. Enterobactin is encoded in the *ent* cluster and is involved with MDR-Kpn strains [28,29]. Yersiniabactin is encoded in the mobile genetic element named ICEKp and plays a secondary role in decreasing the host immune response through evasion of lipocalin 2 [25]. Salmochelin and Aerobactin are encoded in the *iucABCDiutA* and *iroBCDN* clusters carried by the plasmid pLVPK, and their expression is associated with the hypervirulent profile in hvKpn strains [30].

Type VI Secretion System (T6SS) is another important virulence factor that Kpn has developed to translocate proteins, called effectors, into adjacent eukaryotic and prokaryotic cells. Although T6SSs are well characterised in other Gram-negative bacteria, information regarding T6SS function in Kpn remains controversial [31]. Nevertheless, Kpn T6SS is likely to provide an advantage for survival during bacterial competition, such as competition among microbiome bacteria [32]. In addition, this system has been associated with antibiotic resistance, biofilm formation, and the delivery of toxins into neighbouring cells [33].

Toxins are other virulence factors that contribute to virulence enhancement. Colibactin, encoded by the *pks* genomic island, is a Kpn toxin involved in DNA double-strand breaks, chromosome aberrations, and cell cycle arrest [34].

## 3. *Klebsiella pneumoniae* and Public Health

Kpn is the most frequent nosocomial opportunistic pathogen that infects critically ill and immunocompromised patients. This pathogen has become a severe public health problem due to its high prevalence and mortality rate in hospitals [35]. Many reports describing Kpn disease have focused on adults. Nevertheless, recent studies indicate that children (5.9–67.6%) are susceptible to infection by hvKpn strains [36,37].

As previously mentioned, the diseases produced by Kpn include bacteraemia, liver diseases, pneumonia, urinary tract infections, and septic arthritis [38,39,40]. Furthermore, it is one of the most important opportunistic pathogens to combat during solid organ transplants [41,42]. Haematological malignancies, antibiotic administration, mechanical ventilation, and long-term hospitalisations are the principal factors that favour Kpn dissemination in hospitalised patients [43,44,45]. Interestingly, the transmission of Kpn disease during the SARS-CoV-2 pandemic was significantly reduced, along with the transmission of *Enterococcus faecium*, *Staphylococcus aureus*, *Acinetobacter baumannii*, *Pseudomonas aeruginosa*, and *Enterobacter* spp. (called ESKAPE pathogens) [46,47]. Government-implemented public health measures, such as using face masks and gloves, meticulous hand cleaning, access and movement restrictions within the hospitals, and continuous surface cleaning, could be causes of the low incidence of ESKAPE pathogens [48]. Even though antibiotic therapy is ineffective against SARS-CoV-2 infection, some studies reported that 70% of hospitalised patients received broad-spectrum antibiotic therapy [49]. Furthermore, a recent mathematical-method-based study analysed the impact of health interventions on the prevalence of resistant Kpn strains; in that study, an increase in extensively drug-resistant strains was reported, with the frequency rising from 10% to 50% [50].

Genomic studies and sequence type (ST) analysis have suggested that ST307 and ST147 strains have a high number of virulence factors (antibacterial resistance genes, virulence factors, and fitness); these factors are considered a potential risk because they may contribute to the adaptation of these strains to hospital environments and the human host [51]. Kpn ST307 was described in the Netherlands in 2008; however, after five years, the clone was also reported in hospitals in the United States, Pakistan, Colombia, Italy, South Korea, and Tunisia [52]. After 2016, Kpn ST307 was dispersed worldwide, likely as a result of acquiring a second capsule cluster, a fimbrial cluster, and T6SS [53].

The Kpn ST147 clone was first reported in 2008–2009 in Hungary and Spain and is mainly resistant to the fluoroquinolones due to *gyrA* S83I, *parC* S80IQRDR, and *bla*_CTX_-M-15 mutations [54]. From 2011 to 2013, the clone was identified in Greece, Italy, Sweden, Denmark, Canada, the United Kingdom, Finland, India, and Libya. Finally, in 2014, this clone achieved worldwide dispersion [52].

The impact of this uncontrolled antibiotic administration was a subsequent increase in resistant bacterial infections, highlighting the importance of tighter control in prophylactic practice and antibiotic administration. Thus, increasing efforts are being made to develop innovative, specific, and effective treatments to combat the severe and often deadly infections produced by Kpn strains.

## 4. Antibiotic Resistance

**Figure 4:**
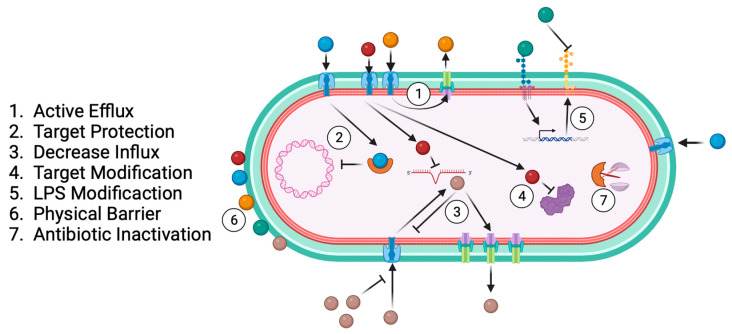
Common bacterial antibiotic resistance mechanisms.

Antibiotic resistance results from bacterial specialisation across time and exposure to different drugs as a bacterial survival strategy [55,56,57]. The main antibiotic-resistance-associated mechanisms are active efflux, target protection, decreased influx, target modification, LPS modification, physical barrier, and antibiotic inactivation (Figure 4).

Efflux pumps are components in bacterial membranes that can extract antibiotics from the cellular environment, a process called active efflux. It may be specific to one antibiotic or able to act against multiple antibiotics (Table 1). An example is the presence of aminoglycoside and quinolone resistance in Kpn, which are associated with the KpnO pump [58]. Meanwhile, OqxAB is expressed in quinolone-resistant Kpn. The KpnEF pump mediates the transport of aminoglycosides, cephalosporins, rifamycin, polymyxins, and tetracyclines. Worryingly, AcrAB-TolC is responsible for polymyxin resistance [59,60].

Sequestering antibiotic molecules inside the bacteria is another resistance mechanism for preventing attachment to their target; it is called “targeting protection”. This mechanism involves the physical association of a target protection protein, and the interaction does not cause a permanent change in the target or the antibiotic [90]. Quinolones form a complex with type II topoisomerases to inhibit the synthesis of bacterial DNA, providing resistance to infection. However, the Qnr pentapeptide repeat protein adopts a structure mimicking B-form DNA and binding to topoisomerase [91]. Kpn harbouring a *qnr* plasmid exhibited the MDR phenotype [92]. In addition, target modification is another mechanism of antibiotic resistance. Mutation of DNA gyrase (*gyrA-gyrB* subunits) or subunits of topoisomerase IV (*parC-parE*) is the principal cause of quinolone resistance [74]. A mutation of DNA gyrase (commonly in *gyr*B) is responsible for nalidixic acid resistance [56]. Modifying the lipopolysaccharide (LPS) structure is a mechanism bacteria develop to affect the target [93]. In the presence of some antibiotics, such as polymyxin, bacteria activate a lipopolysaccharide modification system that involves altering the LPS maturation proteins and incorporating new chains. The Kpn genes responsible for this phenotype are *lpxM*, *pmrE*, and *pafP*, which are involved in lipid maturation [81,82,83].

Antibiotic inactivation is an enzyme-based process where bacterial enzymes modify antibiotic activity by hydrolysis or redox modification, beta-lactamase being the most representative example of this mechanism [57].

Many hydrophilic drugs use porin channels to penetrate bacterial membranes. The regulation of efflux-pump expression is a strategy some bacteria use to limit penetration by these drugs [57]. Efflux pumps such as KdeA, AcrAB, and OqxAB are essential for controlling cell permeability and are involved in quinolone resistance [77,94,95].

Additionally, physical barriers such as capsules and biofilms affect antibiotic diffusion and provide the pathogen with resistance [96]. The antibiotic resistance mechanisms of Kpn highlight the relevance of developing new therapies that could complement or even substitute for antibiotic treatments.

## 5. Treatment Development

A severe health problem in many countries is the administration of antibiotics without susceptibility tests, resulting in high rates of antibiotic-resistant strains [97,98]. Now, in a “post-antibiotic era”, new treatment options are imperative.

Immunotherapy is one of the most promising strategies for treating antibiotic-resistant pathogens, including MDR-Kpn strains. Passive immunisation using Kpn proteins has effective results. However, the high variability of CPS proteins is a limitation of this type of treatment [99]. Another treatment strategy is to use bacteriophages as Trojan horses that cause bacterial lysis. The *Caudoviricetes*, *Myoviridae*, *Siphoviridae*, *Podoviridae*, and *Ackermannviridae* families have been identified to infect the Kpn strain [100]. Mechanistically, bacteriophages create pores in the cell membrane and degrade peptidoglycan, disrupting cell membrane integrity [101]. Intraperitoneally administered in a murine model, bacteriophages showed promising results for therapeutic and prophylactic use against Kpn disease [102,103]. The limitations of this therapy include microbiome alteration and high production costs, and the safety implications in humans are still under investigation. Nevertheless, preliminary findings have shown effective results from the use of combined therapies with antibiotics and bacteriophages against Kpn strains [104].

A priority in the effort against MDR bacteria is developing new efficient therapies or complementing existing treatments. Plant-origin components (POCs) have received particular attention because of their properties, economic viability, and efficacy against bacterial disease. POCs provide an alternative as antibiotic adjuvants, enhancing antibiotic activity.

## 6. The Potential of Plant-Origin Components as a New Treatment Source

Medicinal plants are still used in many societies. The continued use of plants to combat diseases in these societies is based on tradition, economic limitations, and religion. Even though modern medicine has had enormous advances, these sources have been retaken to identify new treatments in the fight against multiple-antibiotic-resistant Kpn diseases.

**Figure 5:**
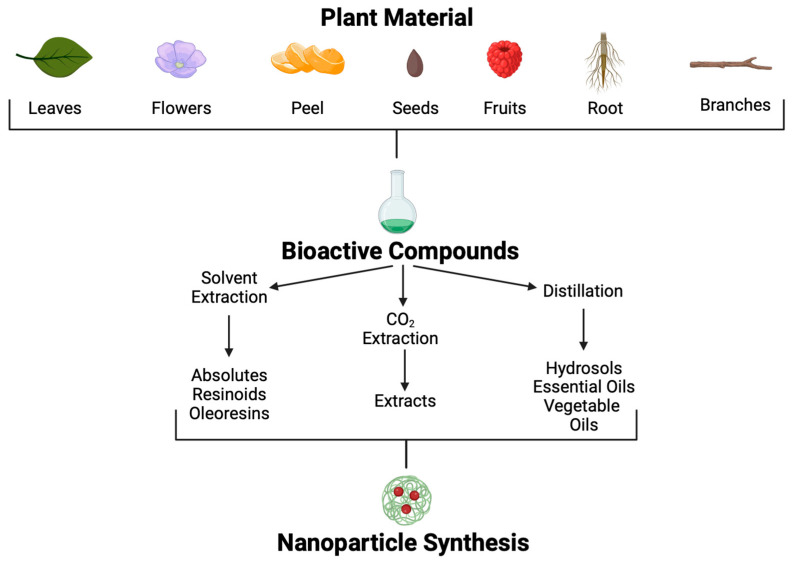
Plant-origin compound extraction methods and application.

Since 35,000 to 70,000 plant species possess therapeutic properties, their use to treat various conditions is a promising approach [105]. Plants produce secondary metabolites in response to environmental factors such as predators, abiotic stress, and interspecific interactions; many of these metabolites could be helpful for pharmaceutic development [106]. Although many POCs and molecules have been isolated, information regarding mechanisms of action, signal transduction, and potential to combat specific diseases is poorly understood. Using different methodological approaches, POCs can be extracted from many parts of plants, such as leaves, seeds, fruits, flowers, roots, wood, etc. (Figure 5).

Different extraction methods are used to obtain these POCs [107]. Through solvent extraction, absolutes, resinoids, and oleoresins can be obtained. Absolutes are aromatic liquids extracted by maceration with chemical solvents, such as ethanol or hexane, followed by filtration and concentration to produce wax. Resinoids are obtained by chemical extraction with solvents from extracts of resinous plant exudates. In contrast, oleoresins are obtained from spice extraction with a hydrocarbon solvent followed by vacuum distillation. On the other hand, aromatic polar and non-polar compounds found in plants at low concentrations, such as essential oils and hydrosols, can be obtained by distillation.

Many plant extracts with antibacterial activity against Kpn strains have been developed (Table 2). For example, ref. [107], *Momordica charantia* leaf ethanolic extract was also efficient against cKpn strains [108], and *Skimmia anquetilia* root extract and *Bacopa monnieri* methanolic and ethanolic leaf extracts were effective against MDR-Kpn strains [109,110].

Moreover, *Paeonia officinalis* showed bacteriostatic activity against MDR-Kpn strains but only when water subfractionation was used, indicating the importance of selecting accurate extraction solvents and subfractionation methods in the search for effective POCs with antibacterial potential [111].

Biofilm formation represents the major challenge in treating Kpn disease, since the construction of this structure restricts penetration by any molecule [129]. Many plant-based products have demonstrated high antibiofilm activity against Kpn, such as *Acacia nilotica* aqueous extract [112], *Himatanthus drasticus* hydroalcoholic extract [113], *Pulicaria crispa* polyphenolic extract [114], *Symplocos racemosa* ethyl acetate extract [115], *Vaccinium corymbosum* water extract [116], and *Vernonia adoensis* chondrillasterol purified from acetone extract [117].

Furthermore, the hypermucoviscosity phenotype of Kpn strains plays an essential role in antibiotic tolerance, as the extracellular matrix, composed of proteins, DNA, lipopolysaccharides, and lipids, acts as a protective barrier that prevents penetration by antibiotics, limiting the design of new therapies. Although many plant extracts have shown excellent results against Kpn, the poor knowledge of biofilms, hypermucoviscosity ecology, and physiology limits the application of these components.

Many antibacterial components cannot cross this barrier and act against the pathogen. Essential oils are secondary metabolites of a lipophilic nature with antibacterial, antiviral, and insecticidal properties, as plants produce them to fight pest invasions and predators [130].

In 2019, Vasconcelos et al. reported that the essential oil obtained from *Origanum vulgare* L. effectively inhibited KPC-Kpn strains *Cinnamomum camphora* essential oil is another plant-origin compound that is effective against MDR-Kpn strains [114]. In addition, essential oils from *Thymus vulgaris* and *Syzygium aromaticum* showed efficiency against KPC-Kpn strains individually and mixed with chitosan to form nanoemulsions, suggesting the use of these treatments to combat brain and central nervous system infections [131].

A combination of natural compounds has also been explored to determine their potential use to fight against Kpn disease. For example, the antibiotic effect of *Melaleuca alternifolia* against KPC-Kpn strains was studied alone as well as in combination with meropenem, amikacin, and colistin, showing that these compounds have a synergistic effect against KPC-Kpn strains [132]. Positive antibacterial results were also observed when using *Cinnamomum burmanii*, *Mentha piperita*, *Thymus vulgaris*, *Camellia japonica*, *Artemisia herba-alba*, and *Thymus algeriensis* essential oils [123,124,125]. Furthermore, combining *M. alternifolia* and *T. vulgaris* essential oils diminished biofilm formation [120]. Our group found that *Thymus vulgaris*, *Mentha piperita*, *Rosmarinus officinalis*, and *Curcuma longa* essential oils effectively against Kpn clinical strains, affecting the hypermucoviscosity phenotype. These findings indicate that essential oils are the best choice to combat mucus production and biofilm-forming strains [121]. However, more studies on essential oils are needed to investigate their safety profile, characterise their mechanism of action, optimise their dose, and observe possible adverse effects in human cells.

In addition to essential oils, bioactive compounds obtained from essential oil extraction were found to have antimicrobial activity. For example, the phenolic monoterpene carvacrol, derived from *Thymus* spp., showed significant inhibitory effects against MDR-Kpn strains in a murine model [133]. Additionally, eugenol treatment affected biofilm formation [134]. The antibacterial effect of cinnamaldehyde and eugenol was demonstrated against KpC strains in vitro and in a murine model [135]. β-Sitosterol isolated from *Kalanchoe tomentosa* and elemicin isolated from *Myristica fragans* acted effectively against MDR-Kpn strains [136,137].

POCs possess a wide range of mechanisms of action against bacteria, such as breakdown of the membrane of the cell, inhibition of wall synthesis, synthesis inhibition and denaturation of proteins, an increase in reactive oxygen species (ROS), alterations to efflux pumps, and the use of metal chelators. Although most POCs’ mechanisms of action against Kpn are insufficiently understood, the mechanisms are well characterised for some POCs. The essential oil of *Juniperus rigida* affects cell permeability and damages the integrity of the membranes of cells, as demonstrated by the leaking of proteins, RNA, and DNA, as well as morphological changes observed using scanning electron microscopy [126]. *Plectranthus amboinicus* essential oil alters membrane integrity and inhibits capsule expression [127]. *Lavandula angustifolia* essential oil and *Cinnamomum verum* oil disrupt bacterial membranes by generating oxidative stress. These oils oxidise the outer membrane, enabling the influx of generated ROS into the cells, causing damage to the cells and eventually death [128,138]. Methanol extract of *Syzygium cumini* has an inhibitory effect on quorum-sensing-regulated violacein production, biofilm production, and exopolysaccharides synthesis [139]. Hexane extract of *Halimeda discoidea* induces different morphological changes during different treatment periods, suggesting that the bioactive compound of the extract has different target sites and killing mechanisms, and their mixture makes the extract effective against multidrug-resistant bacteria [140].

## 7. Biotechnology Applying Plant-Origin Sources to Develop New Control Strategies against Kpn

**Figure 6:**
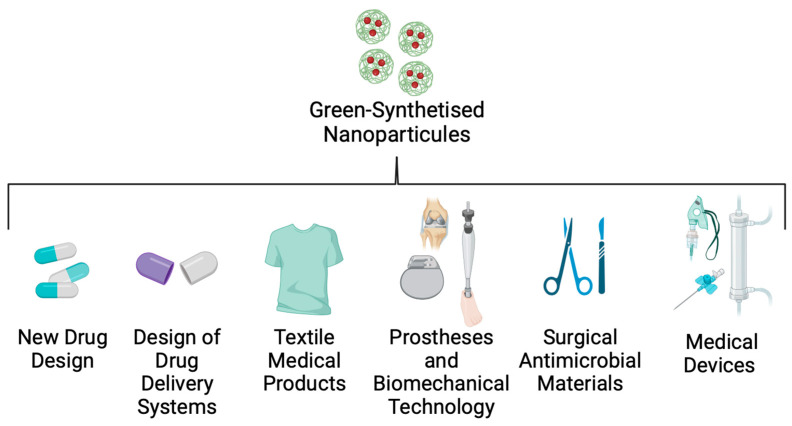
Applications of green-synthesised nanoparticles in biomedicine.

An important factor in controlling and reducing nosocomial infections, such as Kpn disease, is the implementation of new technologies that control intrahospital dispersion of the strains. Many studies have reported that hospital personnel, the environment, and medical devices are the principal routes by which these opportunistic pathogens are transferred to patients [141]. To solve this problem, nanotechnology researchers are working on a nanoparticle (NP) design that could be useful in bionic, textile, and biomedical engineering (Figure 6).

NPs are solid colloidal particles with a 10–1000 nm size range that can offer many benefits [142]. In the biomedical area, NP design focuses on developing new drug delivery materials, photoablation therapy, hospital equipment, and medical clothes that can be valuable tools for controlling the spread of nosocomial diseases [143]. NPs are created by green synthesis technology, which allows the production of these materials to be non-hazardous, eco-friendly, and cost-effective [144]. NPs can be biosynthesised using plant-origin compounds such as extracts and oils (Figure 4).

Metal-binding NPs such as silver (Ag) could bind to bacterial DNA and attach to ribosomes to prevent DNA duplication [145]. Phytochemical-producing plants are valuable as a source of capping agents in AgNP design. These particles have activity as an antibacterial against Kpn strains and exert anti-inflammatory and antioxidant effects in the host. AgNPs from the leaf extract of *Naringi crenulata* are effective against MDR-Kpn strains [146]. Other AgNPs from the leaf extract of *Alternanthera sessilis* have shown potential biomedical application against MDR-Kpn strains [147]. Zinc oxide (ZnSO_4_) NPs produced with *Brassica oleracea* extract showed insufficient antibacterial activity against MDR-Kpn strains but demonstrated antibacterial and antifungal potential against other pathogens [148]. Zirconium oxide (ZrO_2_) NPs are proposed to be functional in the production of medical devices, such as tissue scaffolds, microscale valves, and bone prostheses, suggesting that they possess favourable antibacterial potential. Other ZrO_2_NPs produced with *Phyllanthus niruri* extract have antibacterial properties against several pathogens, including Kpn [149].

Non-metal-binding NP designs are in the process of being studied to identify their antimicrobial potential [150]. Administration of the nonmetal Selenium (Se) is poisonous at higher doses. Nevertheless, SeNPs are gaining increasing attention because of their biomedical effect. Furthermore, SeNPs from *Olea ferruginea* have antimicrobial activity against several nosocomial bacterial strains, including Kpn strains [151].

POCs represent an excellent tool in the development of synergistic therapies to combat infections caused by Kpn. We propose the development of essential-oil-biosynthesised NPs from plants carrying antibiotics against Kpn and other ESKAPE pathogens.

## 8. Special Considerations in the Application of POCs in Medicine

It is important to note that POCs are a complex mixture of secondary metabolites produced by plants, and their chemical composition can change from plant to plant, even within the same species, as a result of exposure to biotic and abiotic factors such as soil hydrology, pH, salinity, temperature, soil organisms, and pollinator insects. Additionally, postharvest treatment, extraction, and conservation methods can influence POCs’ chemical composition.

These results have been the subject of numerous examinations, such as the study performed by Todorova et al. in 2023 [152]. They analysed the chemical composition of commercial and bio-cultivated lavender (*Lavandula angustifolia* Mill.) essential oil. While bio-cultivated lavender essential oil contains 23.13% *β*-linalool, commercial essential oils possess higher amounts (24.34–35.99%). Additionally, only one of the seven samples contained less than 25% linalyl acetate, the content indicated by the requirements of the European Pharmacopoeia. The pharmacological potential of POCs depends on their composition. Establishing them as an official drug could be difficult, and to solve the trouble of composition variations, standardisation of plant growth, postharvest, and extraction protocols is required.

While employing POCs can be among the most effective approaches for developing novel treatments, it is crucial to acknowledge that numerous secondary metabolites synthesised by plants serve as defence mechanisms against infections, insects, and herbivores. These mechanisms function by causing harm to organisms that consume them. Consequently, evaluating the cytotoxic potential of these metabolites is imperative to guarantee their suitability for medicinal plant applications. Although POCs are frequently commended for their aromatic and therapeutic benefits, it is vital to recognise that they can also carry hazards, including the possibility of poisoning. For example, essential oils are highly concentrated plant extracts that can have adverse effects due to the intensity or high concentration of some of their compounds. Recently, many studies have been carried out to characterize this cytotoxicity in vitro and in vivo. Table 3 describes the safety and toxicity data of many POCs that are proposed to have positive effects against Kpn.

## 9. Perspectives

The importance of applied POCs to combat multidrug-resistant infections, such as Kpn disease, lies in the high levels of bioactive compounds that plants naturally produce to defend themselves. Traditional medicine across cultures worldwide can provide insight to identify potential antibacterial compounds that help combat multidrug resistance. Additionally, the synergic effect of these POCs with conventional antibiotic therapies can lead us to design new tools to reduce antibiotic concentrations, administration time, and costs. More research is required to implement nanosynthesis using POCs to develop new antibacterial therapies. Furthermore, it is crucial to identify the toxicity and safety of these products in combating bacterial infections. The complex biology of Kpn represents a limitation in the effectiveness of conventional treatments, and its high dispersion and adaptability require new tools and systems to control and cure the disease it causes. In addition, identifying the mechanisms through which POCs exert their antibacterial effects is a priority. In conclusion, perspectives on designing new therapies based on POCs offer a solution to combat Kpn infections.

**Figure 7:**
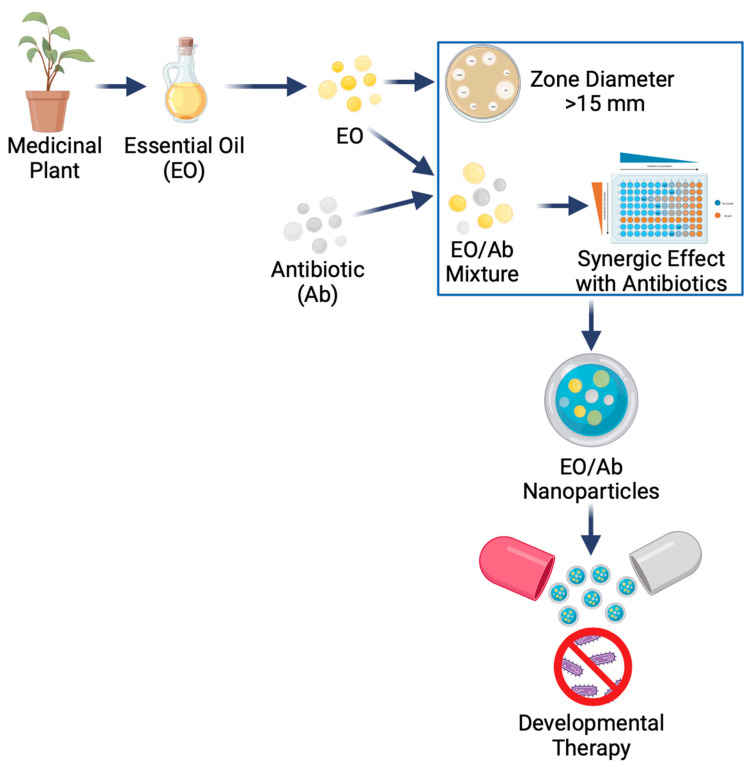
Development of essential oils in the design of new therapies against *Klebsiella pneumoniae*.

Our research group is focused on developing plant compounds that can be employed as synergic therapy with antibiotics (Figure 7). Our last report analysed the potential of essential oils (EOs) against *Klebsiella pneumoniae* infections [121]. We selected EOs with a zone diameter similar to antibiotics’ standard breakpoints (>15 mm) and a synergic effect with antibiotics.

*Thymus vulgaris*, *Curcuma longa*, *Rosmarinus officinalis*, and *Mentha piperita* essential oils were selected from thirty-five EOs, and synergism analysis was performed using the antibiotics gentamycin, ceftazidime, and ciprofloxacin. Interestingly, the EOs showed bactericidal activity comparable to that of antibiotics; nevertheless, the best synergic effect was confirmed for *Thymus vulgaris* with ceftazidime and *Mentha piperita* with gentamycin and ciprofloxacin, regardless of their resistance profile. EOs such as *Cinnamomum zeylanicum*, *Cinnamomum cassia*, *Citrus bergamia*, *Tagetes erecta*, *Aloysia citrodora*, and *Moringa oleifera* essential oils are being evaluated against Kpn strains by our research group. Additionally, our investigation focuses on nanocapsules biosynthesised with EOs and carrying antibiotics.

## 10. Conclusions

The increasing incidence of MDR-Kpn strains in healthcare institutions is a critical threat. Kpn, a nosocomial opportunistic pathogen, affects long-term antibiotic-administered and immunocompromised patients. The increase in pathogens with antibiotic resistance poses a challenge in Kpn disease management, and developing new treatments and strategies, such as immunotherapy and the use of bacteriophages, that help to control these pathogens is important. The antimicrobial potential of plant extracts, essential oils, and biomolecules has recently gained attention. Plant extracts provide low-cost and eco-friendly antibacterial compounds that do not create resistance. New biomedical and bionic engineering techniques are focused on the use of green synthesis of nanoparticles to combat these pathogens. EOs could be a good alternative for bacterial inhibition and synergically used with antibiotics.

Studies are still needed to determine the mechanisms of action of POCs and the toxicity and safety of using them, even though there are currently many studies on their potential to be developed into new treatments. These are essential factors because they will help advance the use of green nanoparticles for biomedical purposes.

## Figures and Tables

**Table 1:** Principal mechanisms and genes related to antibiotic resistance in *Klebsiella pneumoniae*.

Antibiotic	Resistance Mechanisms	Related Genes	Reference
β-Lactams	Antibiotic inactivation	*bla_SHV-2_*	[61]
		*bla_TEM-3_*	[62]
		*bla_CTX-M_*	[63]
		*bla_AMP_*	[64]
		*bla_IMP-1_*	[65]
		*bla_KPC_*	[66]
		*bla_GEN_*	[67]
		*bla_OXA-48_*	[68]
		*bla_VIM-1_*	[69]
	Permeability alterations	*bla_ACT-1_*	[70]
			[71]
Aminoglycosides	Drug modification	*aac*	[72]
		*ant*	
		*aph*	
	Target protection	*armA*	[73]
		*rmt*	[72]
		*npmA*	
	Permeability alterations	*kpnEF*	[59]
Quinolones	Target modification	*gyr*A ^1^	[74]
		*gyr*B ^1^	[75]
		*par*C ^1^	[76]
		*par*E ^1^	
	Permeability alterations	Plasmid-mediated quinolone resistance genes (PMQR)	[77]
	Target protection	*qnr*	[78]
	Drug modification	*aac(6′)-Ib-cr*	[79]
Polymyxin	LPS modification	LPS-MS ^2^	[80]
		*lpxM*	[81]
		*pbgP*	[82]
		*pmrE*	[83]
		*mcr-1*	[84]
		*pmrC*	[85]
		*pagP*	
		*phoPQ*	
		*pmrA*	
		*pmrD*	
	Physical barrier	CPSs ^3^	[86]
Tigecycline	Permeability alterations	*rarA*	[87]
		*ramA*	[88]
		*ramR*	[89]
		*acrR*	
		*rpsJ*	
		*kpgA*	
		*kpgB*	
		*kpgC*	

^1^ Mutations in these genes are responsible for antibiotic resistance; the mere presence of the genes is not. ^2^ LPS-MS = lipopolysaccharide modification system, ^3^ CPSs = capsule polysaccharides.

**Table 2:** Plant-origin compounds that present antibacterial activity against *Klebsiella pneumoniae* strains.

Plant	Type of Compound	Activity	Reference
*Momordica charantia*	Ethanolic leaf extract Ethyl acetate leaf extract	MIC = 625 µg/mL MIC = 156.2 µg/mL	[108]
*Skimmia anquetilia*	Ethyl acetate root extract	Zone diameter = 17.0 ± 1.0 mm MIC = 8 mg/mL	[109,110]
*Bacopa monnieri*	Ethanolic leaf extract Methanolic leaf extracts	Zone diameter =23.0 ± 0.4 mm Zone diameter = 25.0 ± 0.5 mm	[109,110]
*Paeonia officinalis*	Acetone leaf extract	MIC = 128 µg/mL	[111]
*Acacia nilotica*	Aqueous extract	MIC = 11.7 mg/mL MBC = 13.3 mg/mL Reduces biofilm by 59.03%	[112]
*Himatanthus drasticus*	Hydroalcoholic extract	Zone diameter = 16 ± 0.5 mm MIC and MBC = 6250 µg/mL Reduces biofilm by 50%	[113]
*Pulicaria crispa*	Polyphenolic extract	Zone diameter values vary between 12.55 ± 0.31 and 24.00 ± 0.02 mm. MIC values range from 0.1 to 0.425 mg/mL	[114]
*Symplocos racemosa*	Ethyl acetate extract	Zone diameter ranges from 14.33 to 25.66 mm MIC ranges from 0.5 to 10.0 mg/mL	[115]
*Vaccinium corymbosum*	Polyphenolic extract	Reduces the number of attached bacteria and biofilm production by 90% in vitro at 430 µg/mL	[116]
*Vernonia adoensis*	Chondrillasterol purified from acetone extract	Reduces bacterial growth by 38% at 100 µg/mL	[117]
*Origanum vulgare*	Essential oil	Zone diameter = 21 mm MIC = 0.059% (*v*/*v*)	[118]
*Cinnamomum camphora*	Essential oil	MIC = 6.25% (*v*/*v*) MBC = 12.5% (*v*/*v*)	[119]
*Thymus vulgaris*	Essential oil	Zone diameter of 21–30 mm MIC vary from 1 to 16 µg/mL	[120]
		MIC = 0.15% (*v*/*v*) MBC = 0.45% (*v*/*v*)	[121]
*Syzygium aromaticum*	Essential oil	MIC = 0.078% (*v*/*v*) MBC = 0.156% (*v*/*v*)	[122,123,124,125]
*Melaleuca alternifolia*	Essential oil	Zone diameter of 31–40 mm MIC varies from 0.5 to 4.0 µg/mL	[120]
*Cinnamomum burmanii*	Essential oil	MIC = 0.078% (*v*/*v*) MBC = 0.156% (*v*/*v*)	[122,123,124,125]
*Cinnamomum verum*	Essential oil	MIC = 0.5 mg/mL MBC = 1.0 mg/mL	[122,123,124,125]
*Mentha piperita*	Essential oil	Zone diameter of 21–30 mm MIC varies from 8 to 128 µg/mL	[120]
		MIC = 0.60% (*v*/*v*) MBC = 1.25% (*v*/*v*)	[121]
*Camellia japonica*	Essential oil	Zone diameter of 16 mm at 60 µg/mL MIC and MBC = 50 µg/mL	[122]
*Rosmarinus officinalis*	Essential oil	MIC = 0.45% (*v*/*v*) MBC = 3.75% (*v*/*v*)	[121]
*Curcuma longa*	Essential oil	MIC = 2.55% (*v*/*v*) MBC = 6.265% (*v*/*v*)	[121]
*Juniperus rigida*	Essential oil	Zone diameter = 16 ± 0.25 mm MIC and MBC = 3.125 mg/mL	[126]
*Plectranthus amboinicus*	Essential oil	MIC and MBC = 0.08% (700 µg/mL)	[127]
*Lavandula angustifolia*	Essential oil	MIC = 10%	[128]

**Table 3:** Toxicity and safety assessment of some plant-origin compounds that are effective in combating *Klebsiella pneumoniae* infections.

Plant	Model	Activity	Reference
*Momordica charantia*	In vitro in lymphocytes	Lymphocyte viability was 98% at 12.5, 25, and 50 µg/mL), and micronucleus frequency was the same as in the negative control. *M. charantia* extracts did not affect IL-6 or IL-10 production.	[108]
	In vivo in Wistar rats	The acute toxicity test revealed the manifestation of toxic signs in response to the hydroalcoholic extract of *M. charantia*, attributed to the presence of ethanol in the extract. A marginal reduction in body weight, although statistically nonsignificant, was observed. Conversely, administering the aqueous extract did not induce toxic signs or mortality. Both extracts were categorised as class 5, indicating their placement in the toxicity range with an LD50 greater than 2000 mg/kg. In the dermal and ocular irritation test, both extracts were deemed non-irritant.	[153]
*Bacopa monnieri*	In vivo in Sprague–Dawley rats	*B. monnieri* extract (5000 mg/kg) did not cause a histopathological change in the internal organs, including the liver and the kidneys. Rats treated with *B. monnieri* extract at 30, 60, 300, and 1500 mg/kg dosages for 270 days did not present any toxic effect.	[154]
*Paeonia officinalis*	In vivo in Wistar rats	Aqueous extracts of the roots of *P. officinalis* in an acute oral toxicity test did not cause mortality in rats at a dose of 175 mg/kg, 550 mg/kg, or 2000 mg/kg and were considered safe.	[155]
*Acacia nilotica*	In vitro in freshly collected human red blood cells	*A. nilotica* at doses of 50 μg/mL, 100 μg/mL, 150 μg/mL, and 200 μg/mL were found to possess haemolytic activity.	[156]
*Himatanthus drasticus*	In vitro in human erythrocytes and peripheral blood mononuclear cells (PBMCs)	*Himatanthus drasticus* hydroalcoholic extract did not produce significant haemolysis at the concentrations tested, and no significant changes were detected in viability or nitric oxide (NO) production by PBMCs.	[113]
*Symplocos racemosa*	In vivo using Swiss albino mice	In vivo acute oral toxicity testing did not show any toxic effects	[115]
*Origanum vulgare*	In vivo using mice	Continuous use or high doses may deliver undesirable components causing liver and renal function impairment	[157]
*Thymus vulgaris*	In vivo using albino Holtzman rats	While the 28-day oral toxicity test indicated that the no-observed-adverse-effect level (NOAEL) was more than 250 mg/kg/day, *Thymus vulgaris* had moderate oral toxicity.	[158]
*Syzygium aromaticum*	In vitro against human normal dermal fibroblasts	*Syzygium aromaticum* oil cytotoxicity is dose-dependent at a concentration of 0.03%.	[159]
*Cinnamomum verum*	In vivo using *G. mellonella* larvae	*C. verum* leaf EO was non-toxic in the experimental model	[160]
*Mentha piperita*	In vivo and in vitro assays	Several (but not all) short-term and subchronic oral studies noted cystlike lesions in the cerebellum in rats that were given doses of *Mentha piperita* oil containing pulegone, pulegone alone, or large amounts (>200 mg/kg/day) of menthone. Thus, it is safe if the concentration of pulegone in these ingredients does not exceed 1%.	[161]
*Rosmarinus officinalis*	In vivo in Swiss albino mice	No significative changes were reported in relative liver, spleen, heart, or lung size and morphology, and there were changes in clinical chemistry parameters	[162]
*Curcuma longa*	In vivo in Wistar albino rats	No clinical signs of toxicity were observed in any of the treated or control mice at a dose of 5000 mg/kg body weight	[163]

## Data Availability

All relevant data are provided in the manuscript.
